# Assessing Compliance of European Fresh Waters for Copper: Accounting for Bioavailability

**DOI:** 10.1007/s00128-018-2515-1

**Published:** 2018-12-21

**Authors:** Adam Peters, Iain Wilson, Graham Merrington, Dagobert Heijerick, Stijn Baken

**Affiliations:** 1 wca environment ltd. Brunel House, Volunteer Way, Faringdon, Oxfordshire SN7 7YR UK; 2 ARCHE Consulting, Liefkensstraat 35D, 9032 Gent-Wondelgem, Belgium; 3 European Copper Institute, Avenue de Tervueren 168 b-10, 1150 Brussels, Belgium

**Keywords:** Copper, Bioavailability, Environmental quality standard, Water framework directive, Compliance assessment

## Abstract

This study determines the levels of compliance of European fresh waters with a bioavailability-based copper Environmental Quality Standard (EQS). A tiered approach for compliance assessment is used at which the first tier compares the dissolved metal concentration to a threshold, estimated using either regional or continental water chemistry data. At the second tier, the bioavailable metal concentration is calculated using the chemistry of the water body, and compared to the EQS_bioavailable_ for copper. The thresholds at Tier 1 must be set at a level to ensure adequate protection of sensitive environments and to ensure efficient use of regulatory resources. Compliance of 99.3% is observed where bioavailability-based thresholds are used for the implementation derived from regionally relevant water chemistry data. Sites where elevated ambient background levels of copper are combined with high bioavailability (waters with low dissolved organic carbon) are those most likely to be at risk from copper exposures.

The Water Framework Directive (WFD)(2000/60/EC) has specific objectives in regard to restoring all waters to a ‘good’ status through the use of Environmental Quality Standards (EQS) for chemical contaminants. Under the WFD, EQS for some chemicals are set centrally by the European Commission and applied across all Member States, for consistency and comparability. For other chemicals which are deemed to be of more local or specific national issues, the EQS are set and implemented nationally. Copper fits into the latter category (termed Specific Pollutants). This means that each Member State in Europe could potentially have a different EQS for copper.

The importance of accounting for the influence of water chemistry, especially dissolved organic carbon (DOC), in understanding the behaviour, fate and ecotoxicity of inorganic substances has long been recognised in terms of setting environmentally relevant regulatory limit values (e.g. Mance et al. 1984). Biotic Ligand Models (BLMs) have been developed to take account of the effects of metal bioavailability on individual aquatic species (de Schamphelaere et al. 2003; de Schamphelaere and Janssen 2004), and the approaches have been further developed and applied to whole communities (e.g. Peters et al. 2014) although the complexities of the approach have led to difficulties in implementing these models. The specific model which was developed for use in Europe is based on chronic toxicity models and a specific chronic ecotoxicity database (ECI 2008).

Simplified methods for accounting for metal bioavailability, based on the outputs of Biotic Ligand Models (BLMs), for routine regulatory compliance assessment were initially developed by the UK Environment Agency (Environment Agency 2009, 2010). A very similar approach has been adopted in Europe for EQS, for example nickel, that are applied across all Member States. Guidance has been developed on the derivation and implementation of these approaches (e.g. EC 2018; wca 2015). Bio-met, a Microsoft Excel based calculation tool, is a simplified bioavailability tool which uses a simpler set of input parameters, is based on look-up tables compiled from BLM outputs (ECI 2008), and specifically designed for compliance assessment under the WFD (bio-met.net, Baken et al. 2017).

A key aspect of the European approach is that, for regulatory purposes and consistency of implementation across large geographical areas, any agreed EQS for a substance—either set by the EU for all countries or regulated on a national level—will be a ‘fixed’ value, i.e. the same EQS for all waters within the geographic area that it is regulated. Consequently, for substances whose bioavailability (and hence toxicity) is determined by the physicochemical properties of the waterbody, the value of the EQS is set for water chemistry conditions representing high bioavailability. Corrections for bioavailability influenced by the local water chemistry conditions are made to the measured dissolved metal concentrations at the monitoring site. The water chemistry conditions giving ‘high bioavailability’ are determined by using BLMs to derive a Hazardous Concentrations at the 5th percentile (HC5) for all representative water chemistries over a specified area (either national or continental) and selecting the HC5 that would be protective of 95% of all waters in that area (EQS_bioavailable_, EC 2018). Both values, i.e. the bioavailability-based EQS and the calculated bioavailable metal concentration in the water column, are then compared. Effectively this comparison is an assessment of whether the metal in the waterbody being monitored is compliant with (i.e. is less than) the value of the respective metal EQS.

A tiered approach has been used in Europe to assess compliance against an EQS that is bioavailability-based (Comber et al. 2008). The first tier involves comparing the dissolved metal exposure concentration against the EQS which is expressed as “bioavailable metal”, i.e. making the assumption that all of the dissolved metal in the sample is bioavailable. In this paper, we have estimated thresholds for copper using the simplified tool Bio-met. As these thresholds are derived using Bio-met, they are strictly speaking not EQS_bioavailabe_, but for the purposes of the exercise undertaken here, they are used in the same way. Sites can be screened out of the assessment at this tier, if the dissolved metal concentration is below the threshold, or continue to the second tier if the dissolved metal exposure concentration exceeds the threshold. Further information on the water chemistry factors affecting metal bioavailability is required for those sites which remain in the assessment process after the first tier. When applied in this way, the first tier reduces the number of sites at which water chemistry data to support the bioavailability assessment are required. Some countries may choose to collect the water chemistry data (e.g. pH, DOC and Ca) for every monitoring site, but others may prefer focusing such efforts only on those sites which potentially are at risk. Setting an appropriate threshold at Tier 1 is therefore of particular interest to the latter type of countries, since it could considerably limit the cost of monitoring programs.

The aim of this paper is to determine the levels of compliance of European fresh waters with a copper EQS, and to evaluate the usefulness of the regulatory tiered risk-based approach for copper.

## Materials and Methods

Data from two main sources have been used to evaluate the sensitivity of European waters to copper toxicity, and to quantify the magnitude of any potential risks posed to the water environment by copper. Country (i.e. Regional) specific datasets have been obtained from the European Environment Agency (EEA) database for rivers and lakes, Waterbase, (EEA 2016) and from Member State regulatory organisation dissemination portals and personal contacts within regulatory bodies. The second main source of data was for stream waters from the FOREGS database (Salminen et al. 2005). This database has a relatively consistent spatial sampling density throughout Europe (Continental) and provides a consistent dataset over a relatively large scale.

The minimum data requirement for the compliance assessment for copper comprises the input data that required for the user-friendly tool (Bio-met, http://www.bio-met.net) used at Tier 2, which are pH, DOC, dissolved calcium and dissolved copper concentrations. Where dissolved copper concentrations were not available, but pH, DOC and dissolved Ca data of sufficient quality were available; in these cases, total copper concentrations have been used (e.g. for Denmark, Sweden) and can be considered as a worst-case surrogate. Where possible, averages for individual site sample locations with multiple measurements were also derived (as may be used under the WFD). Copper concentrations reported below the detection limit were set equal to the detection limit, representing a worst-case scenario.

The data available from the EEA database (EEA 2016) were filtered to ensure DOC, pH, Ca and dissolved (or total) copper were available for all samples. Data of sufficient quality for the assessment were identified for sites located in Austria and France. Other data sets that were obtained, represented Denmark (provided by the Danish Nature Conservation Agency), Germany (provided by the Rhine Commission), The Netherlands (acquired from the Rhine commission and the Dutch Ministry of Infrastructure and Environment), and Sweden (obtained from the Swedish Agricultural University (SLU)). The number of sites, samples and years covered by monitoring are detailed in Table 1. The sites included from these sources are not necessarily evenly distributed throughout the regions, and may be only broadly representative of the overall range of conditions within a region.

**Table 1:** Summary of the freshwater monitoring datasets, including the number of sites and samples and the thresholds that were derived and used in the assessment

Region	n sites (n samples)	Continental Tier 1 threshold	Regional Tier 1 threshold
Europe	1463 (43,173)	1.6^a^	–
Europe	1463 (43,173)	1.1^b^	–
Austria	85 (6592)	–	1.1
Denmark	41 (379)	–	15.3
France	526 (32,791)	–	2.1
Germany	81 (214)	–	2.3
Netherlands	28 (1587)	–	4.6
Sweden	184 (1092)	–	7.0

^a^Continental threshold set to protect 95% of the waters in the continental dataset

^b^Continental threshold set to protect 95% of the waters in the most sensitive region of the continent

The FOREGS database, representing the continent of Europe, contains 808 samples, but 13 samples have no DOC or pH reported, giving a final dataset of 795 (Table 1). The samples in the FOREGS database for Austria, Denmark, France, Germany, the Netherlands and Sweden were also included in the regional specific datasets. The detection limits were 0.5 mg DOC L^−1^ and 0.08 µg Cu L^−1^. The sampling sites within the FOREGS dataset have a relatively consistent spatial distribution throughout those countries which are covered, but the dataset is based on single spot samples and has a relatively low overall sampling density meaning that country specific resolution is relatively poor (e.g. only 5 samples for Denmark).

The regional and continental datasets were processed using Bio-met to assess the relative sensitivity of the waters to potential copper exposures. The use of Bio-met, rather than the full copper BLM (ECI 2008), to calculate sensitivity was to aid in the processing of thousands of sample data.

The first tier which compares the measured copper exposures to the threshold without accounting for bioavailability is a precautionary screen. Sites which are not screened out at Tier 1 (i.e. where the measured dissolved copper concentration exceeds the threshold) move to Tier 2, at which further information is required about the local water chemistry so that metal bioavailability can be taken into account.

Thresholds were derived from the populations of local HC5 values calculated by Bio-met. Potential thresholds were calculated as the 5th percentile of the distribution of these values. The 5th percentile was selected for consistency with the acceptable error rate for misclassification of water bodies according to an individual EQS (e.g. Crane et al. 2010). Thresholds were derived following three different approaches. The first two approaches both followed a unified continental, approach applying a single Tier 1 threshold (continental threshold) to the entire dataset. Continental thresholds were set using two criteria; (1) at the 5th percentile of the continental dataset, and (2) at the 5th percentile of the most sensitive region (country) across Europe. The latter threshold corresponds to the method for setting the EQS_bioavailable_ for priority substances under the Water Framework Directive (EC 2018).

The third approach included derivation of a region-specific value (regional threshold), only to be used for that country. The regional thresholds were calculated as the 5th percentile of the HC5 values from the datasets for each region. The regional datasets were compiled by combining the FOREGS data for the relevant country with the additional country-specific datasets. This approach for setting the regional threshold is in line with the method for deriving an EQS_bioavailable_ for specific pollutants (EC 2018). Regional threshold values were only derived for those countries for which additional data were available (i.e. the countries listed in Table 2).

**Table 2:** Percentage of sites removed from the compliance assessment process at Tier 1 and Tier 2, using either continental or regional thresholds. The continental threshold of 1.1 µg L^−1^, set to protect 95% of the waters in the most sensitive region, was used for this analysis

Region	Tier 1		Tier 2	
	Continental	Regional	Continental	Regional
Europe	67.2	–	98.9	–
Austria	45.9	45.9	94.9	94.9
Denmark	56.1	100.0	100.0	100.0
France	64.3	94.3	99.4	99.6
Germany	60.5	92.6	98.0	98.7
Netherlands	7.1	96.4	100.0	100.0
Sweden	87.4	99.2	100.0	100.0

## Results and Discussion

**Fig. 1:**
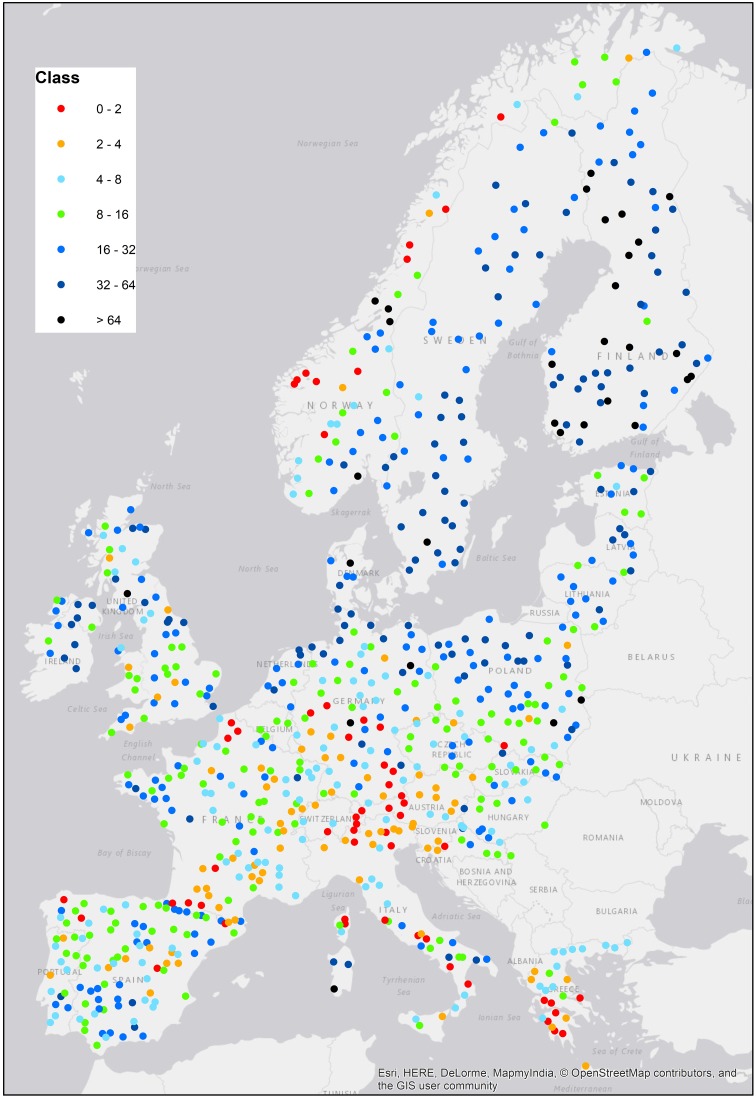
Sensitivity of European surface waters towards copper, from the FOREGS database (Salminen et al. 2005). Coloured points indicate the sensitivity of each site towards copper, as quantified by the thresholds for dissolved copper (µg L^−1^)

Surface water copper sensitivity for both Europe overall and each of the individual countries considered in detail are shown in Table 1. The table shows the continental and regional threshold values which are calculated to be protective of 95% of the site-specific water chemistries and are the basis of the assessment. The spatial distribution of sensitivities of surface waters based on the FOREGS dataset is shown in Fig. 1. The principal advantage of including the FOREGS dataset in the present study is that it is able to reflect differences in sensitivity over broad geographic regions. Based on the FOREGS data, Austria was identified as the region (country) with water chemistry conditions that are the most sensitive to copper exposures. Therefore, in Table 1, the continental threshold identified as the 5th percentile of the most sensitive region is identical to the regional threshold for Austria.

The least sensitive region to copper was Denmark with a threshold of 15.3 µg L^−1^, with the next highest threshold being 7.0 µg L^−1^ for Sweden, and 4.6 µg L^−1^ for The Netherlands. These findings are also confirmed by the spatial distribution of Cu sensitivities in surface waters shown in Fig. 1, which demonstrates that the more sensitive conditions are generally found in mountainous areas such as the Alps and Norway, whereas the coastal plains of Northern Europe are generally rather less sensitive to Cu. On a broad overall level, the two separate sources of data, the FOREGS dataset and the individual country specific datasets, are consistent in terms of the relative sensitivity of the different geographic regions.

The results of Tier 1 screening, using two different approaches towards defining the thresholds (either on a continental scale or a regional scale) are shown in Table 2 as the percentage of sites screened out of the compliance assessment process. Using the continental approach for regions of low sensitivity, such as Denmark and The Netherlands, causes many sites to be taken through to Tier 2. Using the regional, country specific, approach results in fewer sites requiring assessment at Tier 2 for copper, aside from Austria, where many sites are shown to be sensitive and require bioavailability assessment. Considering the number of sampling locations per 1000 km^2^ for each region indicates that whilst Austria, Denmark, and France are relatively well represented, Germany is particularly poorly represented, and The Netherlands and Sweden moderately well represented. Much more detailed datasets would be required in order to adequately represent different water types and ecosystems within a country. Bioavailability conditions vary both spatially and temporally, although as the EQS is expressed as an annual average short to medium term variation is of limited importance to compliance. The FOREGS dataset has a very low spatial sampling density and no replication, and the country specific datasets are not necessarily representative of surface waters in that region.

The most sensitive conditions for copper toxicity in fresh waters occur in mountainous regions near the Norwegian coast line, around the Alps and Pyrenees and in Greece (Fig. 1). The most sensitive conditions are always found where DOC concentrations are low, and these conditions can be very common in mountainous areas with thin soils. The areas surrounding the Baltic Sea generally exhibit much lower sensitivity to copper, and this is largely due to higher concentrations of DOC in the surface waters.

The threshold values would usually be derived from comprehensive datasets and using full, rather than simplified, bioavailability tools. The full BLM tools recalculate the entire ecotoxicity dataset from which the EQS is derived to the water chemistry conditions of interest. The percentile of the dataset which is selected for defining the threshold effectively defines the potential level of under protection by the EQS, because sites which are screened out of the assessment process during Tier 1 do not receive any further consideration (Type I errors). A low threshold ensures that the minimum number of sites are potentially under protected by the threshold (Type II errors), whereas a less stringent threshold can considerably reduce the cost implications of the overall compliance assessment process by reducing the number of sites at which the additional supporting parameters which are required at Tier 2, for the bioavailability assessment to be performed.

The calculated range of sensitivities of the surface waters included in the present study is limited to some extent by a lower limit on the outputs provided by Bio-met, which does not produce threshold values of below 1 µg L^−1^, the generic copper EQS which represents very high bioavailability conditions (Environment Agency 2009), although copper BLM calculations indicate that some highly sensitive waters could have equivalent threshold values which are slightly lower than this value.

The continental compliance assessment applies the same threshold value to all sites and can result in a relatively high proportion of sites continuing to Tier 2 in some regions. The continental threshold which is set to protect 95th percent of the sites in the continental dataset, i.e. at the value of 1.6 µg Cu L^−1^, would not be sufficiently protective of approximately 14% of Austrian surface waters. This is an unacceptable level of potential false negatives (i.e. sites which are removed at Tier 1 which would have failed at Tier 2). This scenario is therefore not further considered in Table 2. Austrian and other alpine waters are amongst the most sensitive to copper in Europe due to their low concentrations of DOC.

It follows that if the same threshold value is to be applied consistently throughout Europe, then a relatively stringent threshold, such as one protecting 95% of all waters in the most sensitive region, would be required in order to ensure adequate protection. However, such a threshold would be ineffective for screening purposes in other regions with less sensitive waters, such as The Netherlands and Denmark. In those regions, a continental threshold has poor ability in discriminating between sites which exhibit no potential risk from those which may potentially be at risk (Table 2).

Provided that the most sensitive region can be identified with reasonable certainty from information which covers the whole area of interest then detailed water chemistry information is only required for that region. The FOREGS dataset includes stream waters from relatively unimpacted locations which have been sampled at a relatively consistent spatial sampling density throughout much of Europe, and provides results which are broadly consistent with those seen from the country specific data in terms of the sensitivity of surface waters to copper. However, whilst broad scale datasets such as FOREGS provide a means to identify sensitive regions they lack the resolution to be able to provide useful information on a regional scale.

Applying regionally defined thresholds in the compliance assessment can potentially result in considerable savings in terms of the additional cost of monitoring to support the refined Tier 2 assessment, which includes an assessment of copper bioavailability (Table 2).

The different approaches to deriving the threshold value, either on an overall continental basis, or on a region-specific basis, highlight the importance of matching the threshold to the local bioavailability conditions. Two distinct situations can be identified which differ from the overall situation: the copper bioavailability conditions in the surface waters of regions may either be particularly sensitive, as is the case in Austria, or particularly less sensitive, as is the case in Denmark. In contrast to Austria, the typical water chemistry conditions in Denmark result in relatively low Cu bioavailability, and the regionally derived threshold value is much higher than those derived for any of the other regions. The other countries are intermediate between these two extremes, with France being more comparable to the Austrian situation, and Sweden and The Netherlands being most similar to the Danish situation. Figure 1 shows a map of Europe for the FOREGS dataset with coloured points indicating the ranges of the regional thresholds for dissolved copper ( µg L^−1^) as calculated by Bio-met. From this figure the regional differences in copper sensitivity are very clear. The most extreme situation, in terms of the implications for the proportion of sites removed at Tier 1, is seen for The Netherlands, where a threshold derived on a continental basis would result in over 90% of sites needing to be processed through Tier 2, whereas a nationally derived threshold would see fewer than 5% of sites needing to be processed through Tier 2.

A total of 11 sites, from an overall dataset of 1502, remained for further assessment after Tier 2. The copper BLM was used to calculate threshold values for each site in order to provide a the most accurate assessment of the sensitivity to copper possible. The threshold values calculated by the copper BLM ranged from 0.6 to 5.5 µg L^−1^, and all of the sites apart from the most and least sensitive had thresholds between 1.2 and 2.0 µg L^−1^. All of the sites in the present study which required assessment at Tier 3 are highly sensitive to copper toxicity.

Risk characterisation ratios calculated for the sites based on the copper BLM were between 0.75 and 6.7. No risk was identified at five of the sites which had proceeded to Tier 3 assessment, which is likely to be due to the slightly precautionary nature of Bio-met. Three of the six sites which failed the compliance assessment at Tier 3 have risk characterisation ratios which are between 1 and 2, and dissolved copper exposures of less than 2.5 µg L^−1^. In these circumstances, failures of the threshold for copper are due to highly sensitive water chemistry conditions combined with slightly elevated ambient concentrations.

Under the EU WFD, the assessment of compliance with an EQS set for the bioavailable metal forms may follow a tiered approach. This requires that the first tier is set at a level which will ensure adequate protection of sensitive environments. The level of the threshold can have important implications in terms of the costs of the compliance assessment on a regional (i.e. country) basis. Setting the threshold at a level where a single value can be considered as sufficiently protective of the whole of Europe would leave some countries with considerable requirements for monitoring water chemistry parameters (e.g. DOC) at the Tier 2 assessment. Deriving thresholds on a region or country specific basis enables the most effective use of resources, without compromising on the level of protection afforded for highly sensitive surface waters. It is likely that further refinement of thresholds could be derived on a more localised geographic basis, although the most important factor in distinguishing between waters with high and low copper bioavailability is whether they are upland or lowland waters, due to the low levels of DOC which are found in the waters of some upland areas due to thin soil cover and low biological productivity, and the more integrative nature of larger lowland waters. Groupings which are based on altitude or soil depth are unlikely to integrate readily with catchment and river basin based systems so are unlikely to be helpful to regulators. The most sensitive conditions for copper toxicity occur when the DOC concentration is low, such as in alpine regions.

Compliance issues for copper are expected to be extremely limited (0.7% of sites considered) if bioavailability based standards are used for the implementation of EQS. Sites where elevated ambient levels of copper are combined with very high bioavailability, principally through very low DOC concentrations, are those most likely to be considered to be at risk due to copper toxicity.

This screening level study demonstrates a clear, resource targeted, approach towards deriving thresholds for assessing compliance against an EQS for copper which is expressed as bioavailable metal, and demonstrates that high levels of compliance are likely to be observed for an EQS for copper which is based on the bioavailable forms.
